# *N*-glucosyltransferase GbNGT1 from ginkgo complements the auxin metabolic pathway

**DOI:** 10.1038/s41438-021-00658-0

**Published:** 2021-11-01

**Authors:** Qinggang Yin, Jing Zhang, Shuhui Wang, Jintang Cheng, Han Gao, Cong Guo, Lianbao Ma, Limin Sun, Xiaoyan Han, Shilin Chen, An Liu

**Affiliations:** 1 Key Laboratory of Beijing for Identification and Safety Evaluation of Chinese Medicine, Institute of Chinese Materia Medica, China Academy of Chinese Medical Sciences, Beijing, 100700 China; 2 Artemisinin Research Center, China Academy of Chinese Medical Sciences, Beijing, 100700 China; 3 Institute of Ginkgo, Pizhou, Jiangsu 221300 China; 4 State Forestry and Grassland Administration Key Laboratory of Silviculture in downstream areas of the Yellow River, College of Forestry, Shandong Agricultural University, Tai’an, 271000 Shandong China; 5 Beijing Botanical Garden, Institute of Botany, Chinese Academy of Sciences, Beijing, 100093 China

**Keywords:** Enzymes, Biocatalysis

## Abstract

As auxins are among the most important phytohormones, the regulation of auxin homeostasis is complex. Generally, auxin conjugates, especially IAA glucosides, are predominant at high auxin levels. Previous research on terminal glucosylation focused mainly on the *O*-position, while IAA-*N*-glucoside and IAA-Asp-*N*-glucoside have been neglected since their discovery in 2001. In our study, IAA-Asp-*N*-glucoside was found to be specifically abundant (as high as 4.13 mg/g) in the seeds of 58 ginkgo cultivars. Furthermore, a novel *N*-glucosyltransferase, termed GbNGT1, was identified via differential transcriptome analysis and in vitro enzymatic testing. It was found that GbNGT1 could catalyze IAA-Asp and IAA to form their corresponding *N*-glucosides. The enzyme was demonstrated to possess a specific catalytic capacity toward the *N*-position of the IAA-amino acid or IAA from 52 substrates. Docking and site-directed mutagenesis of this enzyme confirmed that the E15G mutant could almost completely abolish its *N*-glucosylation ability toward IAA-Asp and IAA in vitro and in vivo. The IAA modification of GbNGT1 and GbGH3.5 was verified by transient expression assay in *Nicotiana benthamiana*. The effect of GbNGT1 on IAA distribution promotes root growth in *Arabidopsis thaliana*.

## Introduction

The plant hormone auxin (indole-3-acetic acid, IAA) was discovered over 70 years ago. Although our understanding of the IAA signaling pathway has thrived in the last few decades, key metabolic pathway enzymes are missing, and the regulation of auxin metabolism is poorly understood^1^. In plants, IAA levels can be attenuated by conjugation (mainly to amino acids and sugars)^2^. IAA conjugates are regarded as either reversible or irreversible storage compounds, and their functions and regulatory genes during plant growth and development are still under investigation^3^.

Amide-linked IAA conjugates (IAA-AA) constitute ~90% of the IAA pool in *Arabidopsis thaliana*^4^. Many enzymes involved in IAA conjugation and hydrolysis have been identified, such as the auxin-inducible GRETCHEN HAGEN3 (GH3) family of amido synthases and various amido hydrolases^2^. Glycosylation can alter the bioactivity, solubility, and cellular localization of aglycones; thus, glycosylation is an important regulatory mechanism for cellular homeostasis and phytohormone activity^5^. Szerszen et al. first cloned an IAGlc synthase cDNA to code an *O*-glycosyltransferase (OGT, IAGLU) from a maize library using antibodies^6^. Subsequently, UGT84B1, UGT74E2, and UGT74D1 were documented to have similar functions to IAGlc synthase in forming 1-*O*-IAA-glucoside in Arabidopsis^7^. When Ljung et al. detected IAA-*N*-glucoside and IAA-Asp-*N*-glucoside in Scots pine (*Pinus sylvestris*), researchers began to recognize a new IAA metabolic branch^8^. Until now, no *N*-glucosyltransferase (NGT) gene has been found, even though two new IAGlc synthases, OsIAGT1 and OsIAGLU, were recently reported to produce IAA-*O*-glucoside in rice (*Oryza sativa*)^9,10^.

NGTs have rarely been identified for small molecules and metabolites, especially compared to OGTs^11^. IAA-*N*-glucoside was thought to be an irreversible form of IAA, since it is more difficult to hydrolyze using hydrolases than IAA-*O*-glucoside^12^. Currently identified NGTs always possess substrate diversity and function as OGTs, *S*-glucosyltransferases (SGTs), or *C*-glucosyltransferases (CGTs). AtUGT72B1 (*A. thaliana*), an NGT, glycosylates toward the OH of 2,4,5-trichlorophenol, the NH2 of 2,3-dichloraaniline, and the SH of 4-chlorothiophenol^13^. MiCGT (*Mangifera indica*) exhibits a robust capability for the stereospecific *C*-glycosylation of 35 structurally diverse drugs, such as scaffolds and simple phenols, using UDP-glucose as a sugar donor. It also forms *O*- and *N*-glycosides^14^. TcCGT1 (*Trollius chinensis*) has been confirmed to catalyze *C*-, *O*-, *N*-, and *S*-glycosylation reactions^15^. The glycosyltransferase specificity of the substrate depends mainly on its structure rather than the primary sequence^5^. Combined with the crystal structure of PtUGT1 (*Polygonum tinctorium*), which could glycosylate the OH of indoxyl^16^, the available structures of the above GTs could be used to explore the structural mechanism of NGTs on IAA.

Herein, we found that IAA-Asp-*N*-glucoside accumulates abundantly in ginkgo seeds (*Ginkgo biloba*); the compound has been reported to inhibit cough^17,18^. The content of IAA-Asp-*N*-glucoside in 58 ginkgo cultivars from China and Japan (two cultivars were introduced from Japan in the 1990s) was 1.02–4.13 mg/g DW higher than that in rice seeds (~0.03 µg/g DW)^19^. By screening candidate GbUGTs from the differential transcriptomes of ginkgo seeds and leaves, we also found that a unique GbNGT1 can perform catalysis at the *N*-position to form IAA-Asp-*N*-glucoside and IAA-*N*-glucoside. Using UDP-glucose as a sugar donor, GbNGT1 specifically glucosylated the *N-*position of IAA-AAs or IAA out of 52 substrates in our enzymatic experiments. Docking analysis and site-directed mutagenesis experiments demonstrated that the E15 residue played a critical role in *N*-glucosylation activity in vitro and in vivo. The in vivo function of GbNGT1 was confirmed with a transient expression assay in *Nicotiana benthamiana*; the effect of GbNGT1 on IAA distribution promotes root growth in *A. thaliana*. These results not only advance knowledge regarding the IAA metabolic pathway but also provide new tools for protein engineering and biosynthetic research.

## Results

### IAA-AA-*N*-glucosides concentrated in *G. biloba* seeds

IAA-AA-*N*-glucosides are compounds present in ginkgo seeds that have been confirmed to be pharmacologically active for cough treatment^17,18^. NMR showed that these compounds, including IAA-Asp-*N*-glucoside and IAA-Glu-*N*-glucoside, were commonly distributed in the mature seeds of 58 ginkgo cultivars or strains (Supplementary Fig. S1 and Table S1). The minimum IAA-Asp-*N*-glucoside content was found in “Dalongyan, Anhui Quanjiao” (1.02 mg/g DW), while the maximum content was found in “No. 18 Xincun” (up to 4.13 mg/g DW). The IAA-Glu-*N*-glucoside content ranged from 0.24 to 1.23 mg/g DW in “No. 1–6, Hubei Anlu” and “No. 2, Guizhou Zhengan”, respectively, and was less than the IAA-Asp-*N*-glucoside content. It is worth mentioning that two Japanese cultivars also had high IAA-AA-*N*-glucoside contents; IAA-Asp-*N*-glucoside and IAA-Glu-*N*-glucoside in “Hisatoshi” and “Jiushou” were 2.51 and 0.63 mg/g DW, respectively; while in “Teng Kuo” or “Tengjiulang”, the contents were 1.88 and 0.55 mg/g DW, respectively.

**Fig. 1:**
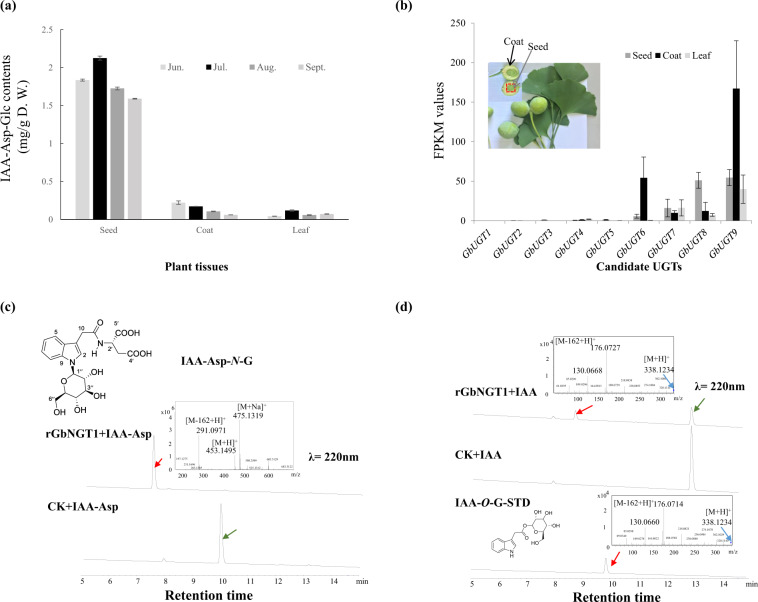
GbNGT1 catalyzed the formation of IAA-*N*-glucoside and IAA-Asp-*N*-glucoside. **a** IAA-Asp-*N*-glucoside content in different tissues at various developmental stages. **b** Transcript levels of nine cloned GbUGTs in samples collected on June 15th of 2018. **c** The new IAA-Asp-glucoside among enzymatic products, as shown by HPLC and MS. **d** The new IAA-*N*-glucoside among enzymatic products, as shown by HPLC and MS. rGbNGT1 is recombinant GbNGT1, CK is the control, and IAA-*O*-G STD is the IAA-*O*-glucoside standard

We analyzed the IAA-AA-*N*-glucoside contents in multiple tissues, including leaves, seed coats, and seeds, at various developmental stages from June 15th to September 15th, 2018 (Fig. 1a and Supplementary Fig. S2). The IAA-Asp-*N*-glucoside content in seeds (ranging from 1.5 to 2.0 mg/g DW) was at least 10-fold higher than that in seed coats (0.07–0.20 mg/g DW) and leaves (0.05–0.13 mg/g DW). The IAA-Glu-*N*-glucoside content span was smaller in the different tissues: seeds had a range of 0.60–0.80 mg/g DW, seed coats were measured at 0.04–0.06 mg/g DW, and leaves were 0.21–0.30 mg/g DW. The IAA-Asp-*N*-glucoside content in seeds and leaves peaked in July, while it gradually decreased in seed coats from June to September. The IAA-Glu-*N*-glucoside content of seeds, seed coats, and leaves reached their maximum values in July, June, and August, respectively.

The presumed precursors of IAA-AA-*N*-glucoside, IAA-AA or IAA-*N*-glucoside, were present in extremely small trace amounts in all tested tissues. These compounds could be only qualitatively detected using UPLC-Q-TOF. The significant difference in IAA-AA-*N*-glucoside contents and their precursors indicates that some UGTs are present in ginkgo seeds and efficiently catalyze the *N*-glucosylation of IAA-AA.

### Identification of ginkgo *N*-glucosyltransferase toward IAA and IAA-AA

Using Arabidopsis UGTs as queries, GbUGTs were screened from the available *G. biloba* genome^20^. The final GbUGT source was UGTs containing the plant secondary product glycosyltransferase (PSPG) boxes of plant UGTs as conserved domains. Combined with differential transcriptome analysis of *G. biloba*, 9 GbUGTs were cloned from 13 candidates (Fig. 1b, Supplementary Table S2 and S3). In the prokaryotic expression experiment (Supplementary Fig. S3a), it was determined that GbUGT8 recombinant proteins were able to catalyze IAA-Asp to produce a new product using UDP-glucose as a sugar donor. Compared to the substrate, the new product’s molecular weight increased by *m*/*z* 162, as detected by mass spectrometry, and the product was further identified as IAA-Asp-*N*-glucoside via NMR analysis (Fig. 1c and Supplementary Table S4). GbUGT8 could not catalyze IAA-Asp to produce new products using UDP-galactose, UDP-glucuronic acid, or UDP-rhamnose as sugar donors. Similar to Asm25 (*Actinosynnema pretiosum*), which catalyzes the in vitro glycosylation of PNDs at the macrolactam amide nitrogen position using UDP-glucose as the sole sugar donor^21^, GbUGT8 could use only UDP-glucose as a sugar donor to catalyze the *N*-glucosylation of IAA-Asp. GbUGT8 was designated UGT717A2 with the help of the UGT Nomenclature Committee, while we used the GbNGT1 representative UGT717A2 as an *N*-glucosyltransferase in the following description.

It was also determined that the recombinant protein could catalyze the *N*-glucosylation of IAA (Fig. 1d). The mass spectrum showed that the new product was an IAA glucoside but not IAA-*O*-glucoside, judging by the standard retention time. The MS product fragments did not contain 218.1234 [M + H-120^+^] or 248.1234 [M + H-90^+^], the characteristic C-glucoside fragments, from the parent ion 338.1234 [M + H^+^] of IAA glucoside^14^. Thus, the product was predicted to be IAA-*N*-glucoside. This product was also found to occur commonly in *G. biloba* seeds (Supplementary Fig. S2). This result indicates that IAA-*N*-glucoside can be produced through the *N*-glucosylation of IAA in plants.

### The specificity of GbNGT1

The enzymatic ability of GbNGT1 at pH 5.0 (138.55 nkat/mg protein), 6.0 (133.56 nkat/mg protein), and 7.0 (129.83 nkat/mg protein) for IAA-Asp was obviously higher than that at pH 8.0 (71.92 nkat/mg protein), which indicates that GbNGT1 could tolerate acid (Supplementary Fig. S4a). Significant differences in enzymatic abilities were not found between 25 °C (81.37 nkat/mg protein) and 35 °C (80.06 nkat/mg protein), whereas the catalytic activity was severely weakened at 45 °C (0.92 nkat/mg protein) and 55 °C (0.37 nkat/mg protein) (Supplementary Fig. S4b). This indicates that GbNGT1 is sensitive to high temperatures. In buffers with metal ions, the enzymatic activity was not significantly changed, indicating that the vitality of GbNGT1 is independent of metal ions (Supplementary Fig. S4c).

**Fig. 2:**
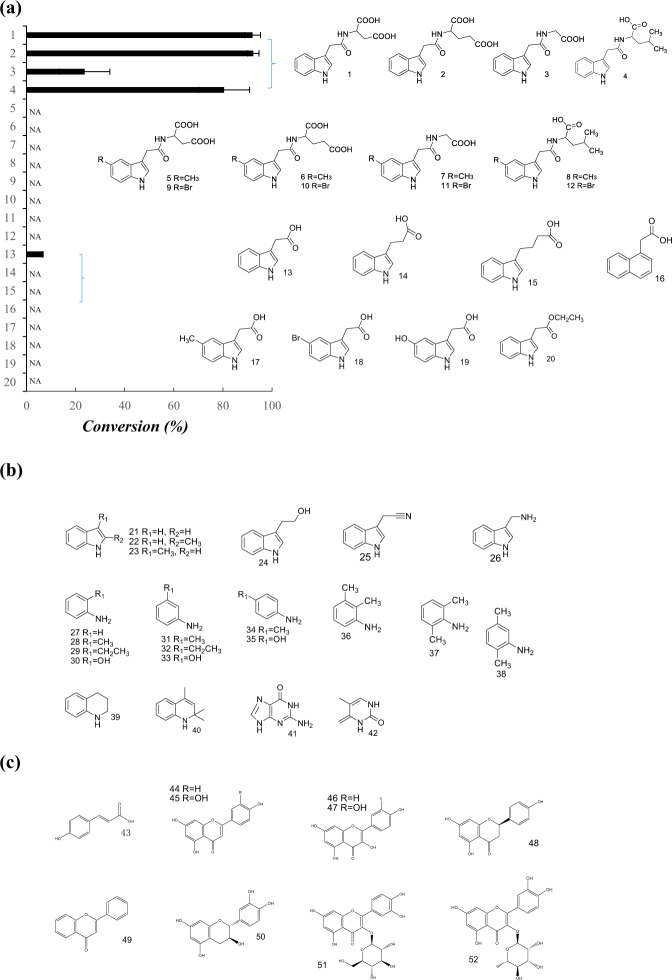
The enzymatic specificity of GbNGT1 as a *N*-glucosyltransferase. **a** The conversion rates of GbNGT1 toward IAA-Asp, IAA-Asp derivatives, IAA, IAA analogs, and IAA derivatives. NA means no product was detected. **b** List of indole amides and anilines that could not be glycosylated by GbNGT1. **c** List of flavonoids that could not be glycosylated by GbNGT1. The compounds used in the enzymatic tests are as follows: IAA-Asp (1), IAA-Glu (2), IAA-Gly (3), IAA-Leu (4), 5-Me-IAA-Asp (5), 5-Me-IAA-Glu (6), 5-Me-IAA-Gly (7), 5-Me-IAA-Leu (8), 5-Br-IAA-Asp (9), 5-Br-IAA-Glu (10), 5-Br-IAA-Gly (11), 5-Br-IAA-Leu (12), IAA (13), IPA (14), indole-3-butyric acid (15), 1-naphthylacetic acid (16), 5-Me-IAA (17), 5-Br-IAA (18), 5-OH-IAA (19), ethyl indole (20), indole (21), 2-Me-indole (22), 3-Me-indole (23), tryptophol (24), indole-3-acetonitrile (25), indole-3-ethylamine (26), aniline (27), O-toluidine (28), 2-ethylaniline (29), O-hydroxyaniline (30), M-methylaniline (31), 3-ethylaniline (32), M-hydroxyaniline (33), P-toluidine (34), P-hydroxyaniline (35), 2,3-dimethylaniline (36), 2,6-dimethylaniline (37), 3,5-dimethylaniline (38), tetrahydroquinoline (39), 2,2,4-trimethyl-1,2-dihydroquinoline (40), guanine (41), thymine (42), p-coumaric acid (43), apigenin (44), luteolin (45), kaempferol (46), quercetin (47), naringenin (48), flavone (49), catechin (50), isoquercitrin (51), quercitrin (52)

Fifty-two substrates were tested to explore whether GbNGT1 possesses the same substrate diversity as UGT72B1, MiCGT, and TcCGT1. MS and NMR verified that only three IAA-AAs could be glucosylated to form their corresponding IAA-AA-*N*-glucosides (Supplementary Fig. S5 and Tables S5 and S6). The converse rates of IAA-Glu, IAA-Gly, and IAA-Leu were 92.3%, 23.7%, and 80.5%, respectively. To investigate the relationship between the substrate electron density and the enzymatic activity, we synthesized various kinds of IAA and IAA-AA derivatives as substrates incorporating a strong electron donor moiety or electron absorption capacity (Supplementary Appendix). GbNGT1 lost its catalytic activity toward IAA-AAs when an electron donor or absorption moieties were added to the benzene ring (Fig. 2a). Similarly, the enzyme could not catalyze modified IAA when the electron donor or electron acceptor group was added. These results demonstrate that GbNGT1 activity was sensitive to electrons and very likely strictly dependent on steric stabilization.

In addition to IAA and IAA-AA derivatives, 25 indole or aniline derivatives were used to test the universal activity of GbNGT1. We found that GbNGT1 could not glucosylate IAA analogs, such as IBA, IPA, and NAA. Furthermore, GbNGT1 could not catalyze aniline or indole derivatives to form new glycosylation products (Fig. 2b), regardless of whether the substitutes (-OH, methyl or ethyl) were near NH2 or N. These results suggest that GbNGT1 is a kind of *N*-glucosyltransferase with strong specificity, which strictly defines the substrate structure; tiny modification or substrate changes could cause steric hindrance and directly alter the enzyme and substrate affinity.

Ginkgo contains various kinds and a great number of flavonoid glucosides; therefore, it was reasonable to test whether GbNGT1 can catalyze flavonoids. Ten flavonoids, including flavone, flavonol, and proanthocyanidin monomers, were used as substrates (Fig. 2c), but GbNGT1 could not catalyze any of these tested flavonoids. These results indicate that GbNGT1 would also be unable to glucosylate the common *C*-position or *O*-position of substrates, which is different from other NGTs.

### The 15th residue Glu (E) determined the *N*-glucosylation function of GbNGT1

Based on the crystal structures of UGT72B1 (PDB No., 2VCH), which glycosylated the *N*-, *S*-, and *O*-positions^13^, we simulated the protein model of GbNGT1, and gain 31% similarity compared to UGT72B1. When using PtUGT1 (5NLM) which glucosylated the OH of indoxyl as reference^16^, we obtain the other model structure of GbNGT1, with 30% similarity toward PtUGT1. The former was selected to imitate molecular docking with UDP-glucose and IAA or IAA-AAs (Supplementary Table S7). Among the five small molecules, the total energy needed for IAA was the highest. This suggested that the enzymatic activity toward IAA would be lower than that toward IAA-AAs, which coincides with our above experimental results (Fig. 2a).

**Fig. 3:**
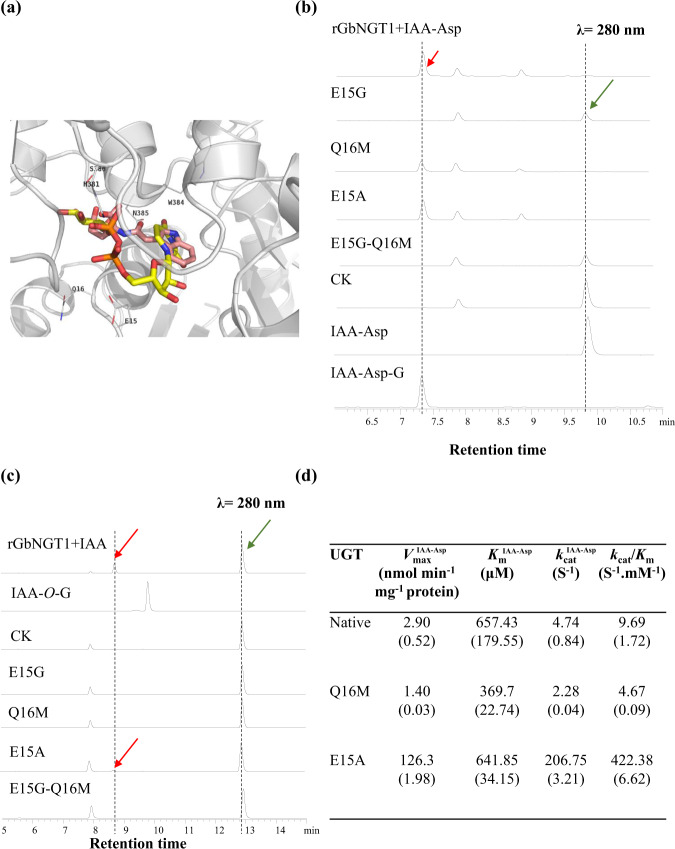
E15 determined the catalytic activity of GbNGT1. **a** The binding domain of GbNGT1 docking with UDPG and IAA-Asp; the molecule in yellow and orange is UDPG; the molecule in pink is IAA-Asp. **b** HPLC chromatograms of enzymatic products, which included native or mutant, UDPG, and IAA-Asp. **c** HPLC chromatograms of enzymatic products, which included native or mutant, UDPG, and IAA. **d** The kinetic data of native and mutants toward IAA-Asp. The molecular weights of recombinant GbNGT1 and mutants with MBP tags were all ~97.72 kDa (MBP tag molecular weight was 42.5 kDa, average was used (SD), *n* = 3. Red arrow, product; green arrow, substrate

The docking results of IAA-Asp, UDP-glucose, and the protein model showed that there were eight key binding substrate residues: His (H381), Trp (W384), Asn (N385), and Gln (Q386) formed hydrogen bonds with UDP-glucose by hydrogen bonds, while Glu (E15), Gln (Q16), Gly (G17), and Gly (G383) formed van der Waals interactions with IAA-Asp (Fig. 3a and Supplementary Fig. S6). Alignments between GbNGT1, UGT72B1, and PtUGT1 showed that the abovementioned residues were identical in position, except for E15 and Q16 (Supplementary Fig. S6).

Four GbNGT1 mutants were obtained by site-directed mutagenesis (Supplementary Fig. S7), including E15G, Q16M, E15A, and the double mutant of E15 and Q16, E15G-Q16M. Enzymatic tests showed that E15G and E15G-Q16M activities were abolished toward IAA-Asp, while E15A and Q16M still possessed different catalytic abilities toward IAA-Asp (Fig. 3b and d). The enzymatic efficiency of E15A toward IAA-Asp was dramatically increased compared to that of native proteins, with the *k*_cat_/*K*_m_ value significantly changed from 9.69 to 422.38 S ^−1^ mM^−1^. The *k*_cat_/*K*_m_ value of Q16M toward IAA-Asp decreased by 4.67 S ^−1^ mM^−1^. These results clarified that the combination of GbNGT1 and its substrates was sensitive to the electron circumstances and steric distribution. This coincides with our conclusions regarding GbNGT1 enzymatic specificity.

For IAA, the enzymatic activities of mutants showed a similar tendency to IAA-Asp; no glucosylation products were detected in the enzymatic experiments with E15G, Q16M, or E15G-Q16M toward IAA. It is worth noting that Ala replaced Glu in E15A, which enhanced its enzymatic efficiency toward IAA-Asp but reduced its enzymatic activity toward IAA (Fig. 3c). These results indicate that the E15 site of GbNGT1 determines its *N*-glucosylation of IAA and IAA-Asp.

### The in vivo function of GbNGT1 in *N. benthamiana*

**Fig. 4:**
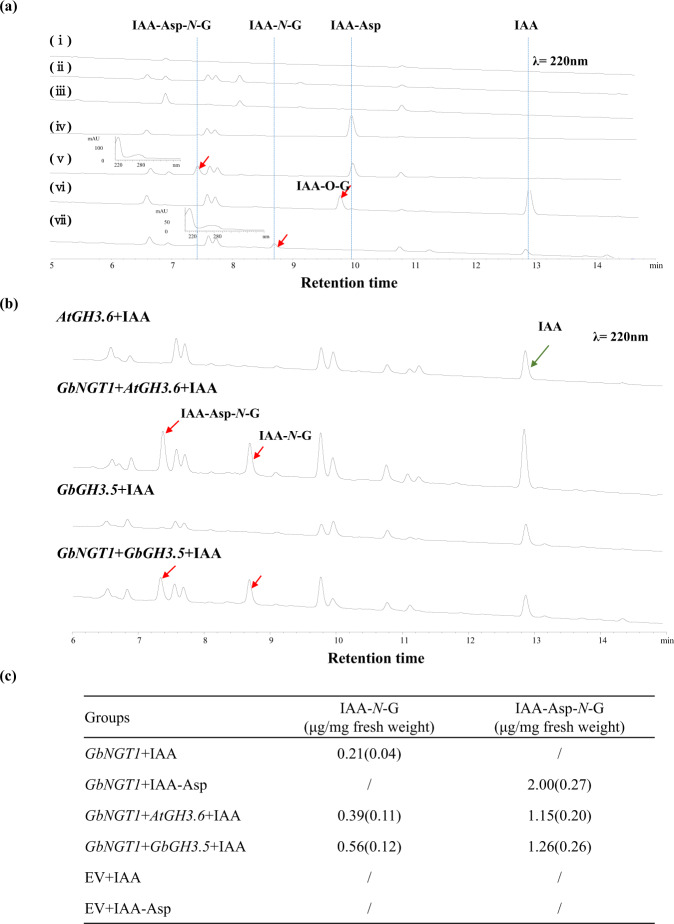
Functional characterization of GbNGT1 in *N. benthamiana*. **a** HPLC spectra of *N. benthamiana* leaves with and without *GbNGT1*, wild type, and empty vector (EV): (i) wild type; (ii) empty vector-transformed leaves; (iii) *GbNGT1*-transformed leaves; (iv) empty vector-transformed leaves adding IAA-Asp; (v) *GbNGT1*-transformed leaves adding IAA-Asp (the inset picture shows the UV spectrum of the product); (vi) empty vector-transformed leaves adding IAA; (vii) *GbNGT1*-transformed leaves adding IAA (the inset picture shows the UV spectrum of the product). **b** GH3s and GBNGT1 reconstructed IAA-Asp-N-glucoside formation in tobacco. **c** The contents of IAA-*N*-glucosides in tobacco leaves transiently expressed different gene combinations; “/” indicates no product detected by HPLC. Averages were used (SD); *n* = 3

The in vivo function of GbNGT1 was tested using *Agrobacterium tumefaciens*-mediated transient expression assays in *N. benthamiana* (Fig. 4a, Supplementary Fig. S3). GbNGT1 coding sequences were cloned under the 35S promoter for the constitutive expression of *N. benthamiana*. Not surprisingly, IAA, IAA-Asp, and IAA-Asp-*N*-glucoside were not detected in the transgenic or wild-type tobacco by HPLC.

Next, IAA or IAA-Asp substrates were fed to the tobacco leaves. The corresponding products, IAA-*N*-glucoside and IAA-Asp-*N*-glucoside, could be found in *GbNGT1*-transformed tobacco leaves but could not be detected in the control. The loss of function of mutant E15G was also verified in *N. benthamiana*, regardless of whether it was toward IAA or IAA-Asp (Supplementary Fig. S7b), which corresponded to the enzymatic results.

*AtGH3.6* was introduced to reconstruct IAA modification in tobacco, since it catalyzes the formation of IAA-Asp from IAA^2^. As expected, both IAA-*N*-glucoside and IAA-Asp-*N*-glucoside could be detected in *AtGH3.6*- and *GbNGT1*-transformed leaves with added IAA. Moreover, 11 candidate *GbGH3* genes were transiently expressed in tobacco with *GbNGT1*; only *GbGH3.5* instead of *AtGH3.6* and the above two IAA *N*-glucosides were detected in the *GbGH3.5*- and *GbNGT1*-transformed leaves (Fig. 4b and c). Interestingly, IAA-*O*-glucoside was found in the control (empty vector-transformed leaves) after adding IAA, while only IAA-*N*-glucoside was detected in the *GbNGT1*-transformed leaves.

### Ectopic expression of *GbNGT1* promotes root growth in *A. thaliana*

**Fig. 5:**
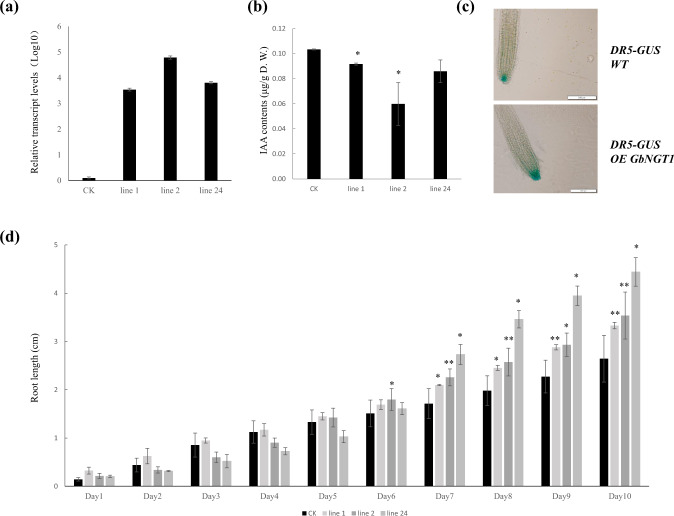
Overexpression of GbNGT1 genes in *A. thaliana*. **a** Relative expression of overexpressed *GbNGT1* in transgenic lines, with *AtPP2A* as a reference gene. Leaves of 5-week-old Arabidopsis were sampled. **b** IAA contents in the aerial parts of 5-week-old Arabidopsis seedlings with CK (wild-type, *col*) or GbNGT1 (lines 1, 2, and 24). **c** GUS staining in the roots of 10-day-old seedlings. The DR5-GUS auxin response reporter was distributed to the root tip of the overexpressed *GbNGT1*. **d** Root length of the transgenic lines 10 days after germination. Values represent the means (SD) of triplicate analytical replicates from independent transgenic lines and CK. Data were statistically evaluated using Student’s *t* test (**P < 0.01, *P < 0.05)

To explore the physiological function of GbNGT1, the genes encoding the protein were ectopically expressed in *A. thaliana*. The transcript levels of the transgenic *A. thaliana* lines overexpressing *GbNGT1* were confirmed by qRT-PCR (Fig. 5a). The total IAA content in the transgenic lines decreased significantly compared with that in the control (Fig. 5b), coinciding with the results for GbNGT1 function in vitro. However, IAA-Asp-*N*-glucoside and IAA-*N*-glucoside were not quantified due to the lack of commercial isotope-labeled standards and traces in *Arabidopsis*.

By observing the growth of roots, we found that root length was obviously longer in the transgenic lines than in the control, particularly on the sixth to tenth day after germination (Fig. 5c). The GUS strain for the auxin response reporter DR5:GUS of the transgenic lines indicated that IAA was distributed to the tip of the root (Fig. 5d). The green fluorescence of DR5:GFP in *GbNGT1*-overexpressing cells showed the same IAA distribution pattern (Supplementary Fig. S8). This result indicates that the overexpression of *GbNGT1* affected root growth in *A. thaliana*.

## Discussion

### Discovery that IAA-Asp-*N*-glucoside enrichment in ginkgo seeds promotes the supplement of the IAA metabolic pathway

IAA is generally found in trace amounts in various forms in plants, including IAA esters (such as IAA-*O*-glucoside) and IAA-AAs^4^. Maize seeds contain 79.5 µg/g DW total IAA, which is the highest ever reported. Oat seeds contain only 8 µg/g DW, while other tested plants contain less than 1 µg/g DW^22^. We found that an amazing amount of IAA-Asp-*N*-glucoside existed in ginkgo seeds, with IAA-Asp-*N*-glucoside contents ranging up to 4 mg/g DW in 58 ginkgo cultivars. This is over one thousand times the amount found in rice seeds (~0.15 µg/g DW), which is the only other plant for which this compound has been reported^19^. Trace amounts of IAA-*N*-glucoside were also detected in ginkgo seeds by UPLC-QTOF. The biosynthetic pathway of IAA-Asp-*N*-glucoside or IAA-*N*-glucoside, also called the IAA *N*-glucosylation pathway, has been totally unidentified until now due to their trace amounts and the limited number of species that contain them, even though these compounds were identified in Scots pine 20 years ago^8^.

**Fig. 6:**
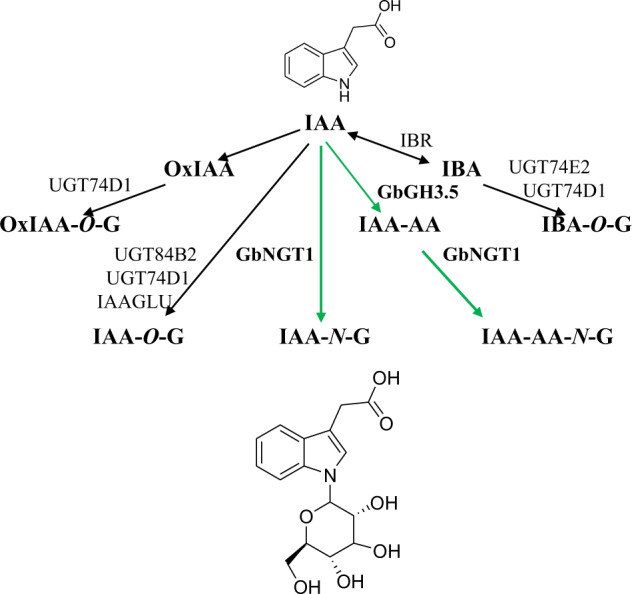
The newly understood IAA metabolism pathway. The character G in IAA-*O*-G or IAA-Asp-*N*-G represents glucoside. The green arrows indicate new branches of the IAA metabolic pathway identified in this study, while the black arrows indicate reported branches in the IAA pathway. IAA-AA indicates IAA-amino acids, including IAA-Asp and IAA-Glu

In this work, we found through large-scale resource screenings that IAA-Asp-*N*-glucoside hyperaccumulates in ginkgo seeds. After differential transcriptome analysis, we first cloned IAA *N*-glucosyltransferase (NGT) and systematically identified the specificity, which completed the metabolic network of IAA. Based on the GbNGT1 enzyme activity toward IAA or IAA-Asp and the widely studied IAA-*O*-glucoside pathway, the formation of IAA *N*-glycoside may occur through one of two mechanisms: (i) amino acids are added to IAA first to form IAA-AA by GH3s and then glucosylated to IAA-Asp-*N*-glucoside by UGTs; alternatively, (ii) IAA-*N*-glucoside is first produced by UGTs by glucosylation of the IAA *N*-position, and then GH3s catalyzes the formation of IAA-Asp-*N*-glucoside (Fig. 6). The transient expression of *GbNGT1* and *GbGH3.5* in tobacco corresponds to the first mechanism; however, there may be differentiation or diversification of GH3s in ginkgo, which needs more GH3 functional exploration.

The known metabolic pathway of IAA includes *O*-glucosyltransferases and amino acid conjugate synthetases. OGTs and GH3s directly affect the form and dynamic equilibrium of IAA in plants and thus regulate plant growth and development^23^. The first reported IAA *O*-glucosyltransferase was IAGLU from maize, which was identified in 1994^6^. This kind of OGT was also found in Arabidopsis, duckweed, cauliflower, soybean, tomato, rice, and tobacco. These OGTs widely affect the structure, growth, and development of plants, such as leaf angle and structure, dwarf, and flower development, by regulating the balance of IAA in plants^5^. With the identification of the GH3 family, which catalyzes the formation of IAA-amino acids in Arabidopsis, the GH3s in rice, moss, peas (*Pisum sativum*), and strawberries were reported to be involved in antipathogen functions, shoot cell elongation, lateral root development, and root geotropism^24–26^. The ectopic expression of *GbNGT1* promotes root growth in *A. thaliana*, in contrast to the function of UGT84B1^27^. This may be caused by the *N*-glucosylation being different from the *O*-glucosylation of IAA. We further observed that the auxin reporter signals (DR5-GUS and DR5-GFP) in the roots of transgenic *GbNGT1* lines were distributed to the root tip, in contrast to the wild type. IAA homeostasis plays a central role in determining root architecture, from the initiation of a root meristem during embryogenesis to the generation of a functional root system with a primary root, secondary lateral root branches, and adventitious roots^28^. Increasing IAA in roots may prompt plants to assign more carbohydrates to the roots, enhance root growth and improve plant resistance to environmental stress such as drought^29^ and flooding^30^.

GbNGT1 not only determines the new IAA metabolic branch but also plays an important role in the downstream modification of the known IAA-AA branch; thus, its function may contribute to the homeostasis of IAA in planta. Per our results, GbNGT1 could affect the distribution of IAA in root tips and may be the cause of increased root growth in transgenic lines compared to the control. The identification of GbNGT1 greatly complements the metabolic network of IAA and opens up new directions for the study of IAA homeostasis in plants.

As the main product of IAA-amino acid conjugates, IAA-Asp was considered to be an irreversible form of IAA and of no use to plants until its hydrolases were found^8^. In Chinese cabbage (*Brassica rapa*), the enzymatic activity of IAA-Asp hydrolysis increased in *Plasmodiophora brassicae*-infected root galls compared with control roots^31^; in *M. truncatula*, MtIAR31, -32, -33, and -34 displayed hydrolytic activities against IAA-Asp and IBA-Ala^32^. At present, IAA-*N*-glucoside is also considered a more stable molecule than IAA-*O*-glucoside^12^; however, the purpose and physiological function of the high IAA-Asp-*N*-glucoside concentrations in ginkgo seeds are still unknown. An additional research question includes whether there are any hydrolases that could cleave the C-N bond to free IAA in ginkgo. These areas need further research in the future.

### Specificity of *N*-glucosylation

*N*-glycosylation modification of proteins or peptides is more common than that of small molecules. Asparagine (Asn) is generally considered to be the main amino acid site for *N*-glycosylation in peptides and is characterized by the covalent attachment of an oligosaccharide to its side chain amide^33^. GbNGT1 was able to glucosylate small molecules, including IAA-Asp, IAA-Glu, IAA-Leu, and IAA-Gly, at the *N*-position of IAA residues but not at the amide nitrogen of their side chains. Our results confirm that the catalytic mode of *N*-glycosyltransferases toward small molecules and proteins is different^34^.

IAA *N*-glucosides exist in monocotyledons (rice and maize) and dicotyledons (Arabidopsis, *Lotus japonicus*)^19^, gymnosperms (Scots pine and ginkgo)^8^ and even in microbes (*Cortinarius brunneu*)^35^. This indicates that the enzymes contribute to biosynthesis and should be conserved. Homologous genes encoding OGT for IAA and NGTs for other small molecules could be easily found in different species. This suggests that UGT amino acid sequences are related to their function in plants. OsIAGLU showed 67% amino acid identity with ZmIAGLU, which possessed the same function of glucosylating the hydroxyl of IAA^10^. Similarly, BnUGT1 and AtUGT72B1 shared 85% sequence identity and the same *O*-glycosylation function toward 3,4-dichlorophenol. In contrast, only AtUGT72B1 could glucosylate the *N*-position of 3,4-dichloraaniline^13^. The highest similarity among GbNGT1 and other UGTs was only 44% from *Picea sitchensis* (GenBank: ABR17691.1) in the NCBI database; moreover, the homologous genes of GbNGT1 could not be found by blast with the default value, even in the ginkgo genome. These results suggest that the catalytic capacity of *N*-glucosyltransferase toward IAA or IAA-AA may be less dependent on amino acid sequence and rely mainly on protein structure.

The reported *N*-glycosyltransferases usually catalyze different types of glycosylation reactions at different sites. AtUGT72B1 was able to conduct the *O*-glycosylation of 2,4,5-trichlorophenol, the *N*-glycosylation of 2,3-dichloraaniline, and the *S*-glycosylation of 4-chlorothiophenol^13^. MiCGT could glycosylate the *C*-position of maclurin, the *O*-position of phenol, and the *N*-position of 3,4-dichloraaniline^14^. TcCGT1 catalyzed four types of glycosylation at the *C*- or *O*-position of phenols and flavonoids, the *N*-position of 3,4-dichloraaniline, and the *S*-position of 3,4-chlorothiophenol^15^. In contrast, GbNGT1 specifically catalyzed the *N*-glucosylation of IAA or IAA-AA.

Generally, the relatively preserved PSPG motif located in the C-terminus of UGTs was bonded to the sugar donor, while the diverse residues close to the N-terminus were bonded to the sugar acceptor, which determined the specific selection of substrates^5^. To date, very few key residues in the N-terminus have been identified, even though some crystal structures of NGTs have been revealed. H24 and E396 of TcCGT1 were found to play a key role in the stabilization and localization of small molecule substrates; in addition, the mutants I94E and G284K of TcCGT1 could convert enzymatic activity from the *C*- to the *O*- position^15^. After converting all five amino acid residues within 314–320 of BnUGT1 to the corresponding key amino acids in UGT72B1, BnUGT1 gained *N*-glycosylation activity toward 2,3-dichloraaniline. The *O*-glycosylation activity of the UGT72B1 mutant H19Q was severely decreased toward 3,4-dichlorophenol, with the *k*_cat_ value reduced to 1/300th of that of the native protein. Simultaneously, the *N*-glycosylation capacity of H19Q toward 3,4-dichloraaniline dropped to 1/2 of that of the native protein^13^. In our study, amino acid alignments revealed that H18 near the N-terminus of GbNGT1 corresponded to the critical sites H19 in AtUGT72B1 and H24 in TcCGT1. Docking analysis identified another special residue, E15, that binds to IAA-Asp in GbNGT1. Mutating E15 (E, glutamic acid) to E15G (G, glycine) drastically decreased or even abolished the catalytic activity of *N*-glycosylation toward IAA-Asp or IAA in vitro and in vivo, whereas converting E15 to E15A (A, alanine) enhanced the enzymatic efficiency toward IAA-Asp by 40-fold compared to the native protein. Thus, E15 is another critical N-terminus residue determining the specificity of substrates in addition to His in the N-terminus of GbNGT1, which provides a new reference site for the reconstruction of NGTs.

### The application prospects of GbNGT1

Ginkgo flavonoids and ginkgolides have been widely used as important drug and dietary supplements^36^. IAA-Asp-*N*-glucoside has been discussed as the main pharmacological cough-inhibiting compound in ginkgo and can also be used as an anti-asthmatic and for phlegm elimination^17,18^; furthermore, considerable pharmacological studies on IAA derivates and indole alkaloids have shown IAA-induced cell death in combination with UV-B irradiation to increase apoptosis in PC-3 prostate cancer cells^37^. Indole-*N*-glucosides have the potential to serve as novel SGLT2-selective inhibitors (sodium-glucose cotransporters) to cure type II diabetes^38^. This suggests that IAA derivates have the potential for pharmaceutical development. GbNGT1 not only has high specific activity toward IAA-AA but also possesses spatial specificity, indicating that these enzymes have potential uses in engineering the biosynthesis of IAA-AA-*N*-glucoside.

## Materials and methods

### Materials and chemicals

Ginkgo leaves, seed coats, and seeds at different developmental stages from June 15th to September 15th were collected in Beijing botanical gardens. Mature seeds from 58 cultivars were collected in October at the Pizhou Resource Nursery (Jiangsu, China). These samples were immediately frozen in liquid nitrogen and stored at −80 °C for further use. The substrates tested in this study were purchased from Xili Limited Co. (Shanghai, China) and Indofine (Hillsborough, NJ, USA). UDP-glucose, UDP-galactose, and UDP-glucuronic acid were purchased from Sigma-Aldrich (Oakville, CA, USA). UDP-rhamnose was enzymatically synthesized using methods published by Rautengarten et al.^39^. All chemicals used in this study were of analytical or HPLC grade.

### Analysis of IAA-AA-*N*-glucoside metabolites by HPLC

A 50 mg dry weight sample was extracted with 2.5 mL of methanol (25%) and then placed in an ultrasonic bath at 25 °C for 30 min. The supernatant was filtered through a membrane with a pore diameter of 0.22 µm after 10 min of centrifugation (12,000 rpm) at 4 °C. Finally, a 10 µL aliquot was injected for subsequent analysis. HPLC was used to determine the components based on the chromatographic separation terms at a wavelength of 280 or 220 nm.

Chromatographic separation was achieved using a Venusil Innoval C18 (250 mm × 4.6 mm, 5 μm), The column temperature was maintained at 30 °C, and the autosampler temperature was set at 4 °C, with 0.1% formic acid in water and acetonitrile as solvents A and B. The injection volume per sample was 10 μL, and the flow rate was 1.0 mL/min. Elution conditions were as follows: 0–30 min, B from 5 to 100%; 30–35 min, B from 100 to 100%.

### RNA-seq, candidate gene sequence analysis, and gene clone

In order to examine the expression patterns of the GbUGT and GbGH3s genes associated with the IAA-*N*-glucoside and IAA-AA-glycoside biosynthetic pathways, RNA was sequenced from leaves and seeds at different stages of development using an Illumina HiSeq2000 platform. RNA extraction, sequencing, and read filtering were performed as previously described by Yin et al.^40^. Finally, 13 GbUGT genes and 11 GbGH3 genes were selected according to the published genome and transcriptome of *G. biloba*. Multiple sequencing alignments of the deduced amino acid sequences were carried out using DNAMAN. The predicted amino acid sequences of UGTs and GH3s were aligned using Clustal X2. Mixed cDNAs from leaves and seeds of *G. biloba* were used for gene amplification. For GbUGTs, the PCR products were purified and digested using the corresponding restriction enzymes, ligated to a pMAL-c2x vector (New England BioLabs, Ipswich, MA, USA), and digested with the same restriction enzymes used for the expression of recombinant proteins in *Escherichia coli*. For the *GbGH3s* and *AtGH3.6* genes, a gateway system was used to construct the pK7WG2D vector for verification of IAA modification in planta.

### Enzyme assay and product identification

Purification of recombinant UGT proteins in *E. coli* and enzymatic activity tests were performed with minor modifications as previously described (Yin et al.)^41^. In the enzymatic tests and kinetic analysis of the recombinant GbUGT proteins, purified enzymes (1–2 µg) were incubated in reaction mixtures comprised of 10 mM DTT, 50 mM Tris-HCl (pH 7.0), and 2 mM crude UDP-glucose, UDP-glucuronic acid, UDP-galactose, or UDP-rhamnose (20 µL enzymatic crude solutions containing UDP-rhamnose) in a final volume of 50 µL. The concentration of the tested acceptor substrates ranged from 100 to 2000 µM. Reactions were stopped by adding methanol after 30 min of incubation at 37 °C. The samples were centrifuged at 14,000 rpm for 10 min at 4 °C and further analyzed with HPLC using the abovementioned procedure. The kinetic parameters *K*_m_ and *k*_cat_ were calculated using the Hyper 32 program (http://hyper32.software.informer.com/).

The enzymatic reaction solution was filtered through 0.22 µm membranes. A 10 µL aliquot was used to analyze any new products with HPLC, and then a 1 µL aliquot was injected into UPLC-MS/MS to identify the products. UPLC-QTOF-MS/MS detection used a 6540 Agilent 1260 photodiode array. Electrospray ionization (ESI) was applied in positive (PI) mode for MS and MS/MS to collect fragment information for the molecular weights. The positive mode parameters were optimized as follows: HV voltage, 3.5 kV; capillary, 0.095 μA; nozzle voltage, 1500 V; gas flow, 8 L min^−1^; gas temp, 320 °C; nebulizer, 35 psi; sheath gas temp, 350 °C; sheath gas flow, 12 L min^−1^; and scan range, *m*/*z* 50–1250 units. A collision energy of 15 V was used during MS/MS analysis.

### Homology modeling and docking statistics

Homology models of GbNGT1 were built using the crystal structures of UGT72B1 (PDB No., 2VCH) and PtUGT1 (5NLM) as templates on the Phyre2 server at http://www.sbg.bio.ic.ac.uk/phyre2^42^. UDP-Glc and sugar acceptors (IAA, IAA-Asp, IAA-Gly, IAA-Glu, or IAA-Leu) were docked with the model structure of GbNGT1 from UGT72B1 using the igemdock 2.1 program. The models were visualized with the PyMOL molecular graphics system at http://www.pymol.org.

### Functional characterization of GbNGT1 in *N. benthamiana* and *A. thaliana*

*GbNGT1*, mutant *E15G*, *AtGH3.6* (F24B18.13, At5g54510), and *GbGH3s* sequences were subcloned into the binary vector pK7WG2D (with GFP) using the Gateway LR protocol. The *A. tumefaciens* clone GV3101 clone containing the *GbNGT1, GbGH3s, AtGH3.6*, or *E15G* genes, was incubated in 50 mL of LB culture containing 50 mg L ^−1^ spectinomycin and 50 mg L ^−1^ rifampicin overnight (200 rpm, 28 °C). The culture was centrifuged for 10 min at 3000 *×* *g*. The *A. tumefaciens* pellet was resuspended and washed with infiltration buffer (10 mM MES, 10 mM MgCl_2_, and 100 μM acetosyringone). The bacterial solution was adjusted to a final OD_600 nm_ = 0.5. The transient transformation assay was conducted through leaf infiltration on 4-week-old *N. benthamiana* plants growing under a 16/8 h light/dark rhythm. LB 985 NightShade (Berthold Technologies) and OLYMPUS IX73 were used to determine whether *GbNGT1*, *E15G*, and *AtGH3.6* were transiently expressed in tobacco leaves by GFP signal (Fig. S2). After infection for 24 h, the substrates IAA (4 mM) or IAA-Asp (4 mM) were infiltrated into the leaves with the transformed genes of *N. benthamiana*. After 36 h of substrate injection, the infected leaves were harvested, weighed (100–200 mg), ground, and then extracted with a 500 μL methanol solution (V_methanol_:V_water_ = 7:3). After sonication for 30 min, the samples were centrifuged at 12,000 rpm and filtered through membranes with a pore diameter of 0.22 µm. Finally, a 10 µL aliquot was applied for HPLC quantitative analysis, and 1 µL samples we used for UPLC-QTOF qualitative analysis.

*GbNGT1:* pK7WG2D (without GFP) was transformed into *A. thaliana* via GV3101. Kanamycin was used to screen the transgenic lines. RNA from transgenic *A. thaliana* lines overexpressing *GbNGT1* was extracted using an RNAprep Pure Plant Kit (Tiangen Biotech Co., Beijing, China), followed by reverse transcription after the removal of genomic DNA contamination. The *P2PA* gene was used as a housekeeping gene for these qRT-PCRs. Seeds from the transgenic lines and wild type (CK) were grown on 1/2 MS medium in darkness for 3 days at 4 °C for vernalization, then transferred to illumination at 25 °C and vertically placed to record root length. ImageJ was used to measure the root length. Homogeneous transgenic *proDR5-GFP* and *proDR5-GUS* Arabidopsis plants were hybridized with *GbNGT1* overexpression lines with a Col genetic background.

### Analysis of IAA in Arabidopsis

Arabidopsis seedlings (5 weeks old) were freeze-dried and ground into powder. Then, a 25 mg aliquot of each individual sample was precisely weighed and transferred to an Eppendorf tube. After the addition of 1000 μL of extraction solution (50% acetonitrile in water, precooled at −40 °C, containing isotopically labeled internal standard mixture), the samples were vortexed for 30 s, sonicated for 5 min in an ice-water bath, and homogenized at 40 Hz for 4 min. The homogenization and sonication cycle was repeated twice. After centrifugation (10 min, 12,000 rpm, and 4 °C), an 800 μL aliquot of the supernatant was further purified with SPE. The SPE cartridges were washed with 1 mL of methanol and then equilibrated with 1 mL of 50% ACN/H2O (v/v). After loading a sample (supernatant obtained following the procedure described above), the flow-through fraction was discarded. The cartridge was then rinsed with 1 mL of 60% ACN/H2O (v/v). After this single-step SPE, the samples were evaporated to dryness under a gentle stream of nitrogen and reconstituted in 100 μL of 10% ACN/H2O (v/v). All the samples were vortexed for 30 s, homogenized at 60 Hz for 4 min, and sonicated for 5 min in an ice-water bath. After centrifugation (10 min, 12,000 rpm, and 4 °C), the clear supernatant was subjected to UPLC-MS/MS analysis.

UPLC separation was carried out using an EXIONLC System (Sciex) equipped with a Waters ACQUITY UPLC CSH C18 column (150 × 2.1 mm, 1.7 μm, Waters). Mobile phase A was 0.01% formic acid in water, and mobile phase B was 0.01% formic acid in acetonitrile. The column temperature was set at 50 °C. The autosampler temperature was set at 4 °C, and the injection volume was 5 μL. A SCIEX 6500 QTRAP + triple quadrupole mass spectrometer (Sciex) equipped with an IonDrive Turbo V electrospray ionization (ESI) interface was applied for assay development. Typical ion source parameters were curtain gas = 40 psi, ion spray voltage = ±4500 V, temperature = 475 °C, ion source gas 1 = 30 psi, and ion source gas 2 = 30 psi. The MRM parameters for each of the targeted analytes were optimized using flow injection analysis by injecting standard solutions of the individual analytes into the API source of the mass spectrometer. Several of the most sensitive transitions were used in MRM scan mode to optimize the collision energy for the 176.0, 130.0/176.0,103.0 pair. SCIEX Analyst Work Station Software (Version 1.6.3) and Sciex MultiQuant™ 3.0.3 were employed for MRM data acquisition and processing.

### Statistical analysis

Statistical analyses were performed using Excel (Microsoft Office, Microsoft). *P*-values were calculated using an unpaired, two-sided Student’s *t* test (**p < 0.01; *p < 0.05). Data are presented as the mean (standard deviation) (*n* ≥ 3).

## Supplementary information

Revised Supplementary information

## Data Availability

All data supporting this research result can be obtained in the paper and within its Supplementary Materials published online.
