# Correction to: *N*-glucosyltransferase GbNGT1 from ginkgo complements the auxin metabolic pathway

**DOI:** 10.1093/hr/uhac065

**Published:** 2022-03-18

**Authors:** Qinggang Yin, Jing Zhang, Shuhui Wang, Jintang Cheng, Han Gao, Cong Guo, Lianbao Ma, Limin Sun, Xiaoyan Han, Shilin Chen, An Liu

**Affiliations:** 1 Key Laboratory of Beijing for Identification and Safety Evaluation of Chinese Medicine, Institute of Chinese Materia Medica, China Academy of Chinese Medical Sciences, Beijing 100700, China; 2 Artemisinin Research Center, China Academy of Chinese Medical Sciences, Beijing 100700, China; 3 Institute of Ginkgo, Pizhou, Jiangsu 221300, China; 4 State Forestry and Grassland Administration Key Laboratory of Silviculture in downstream areas of the Yellow River, College of Forestry, Shandong Agricultural University, Tai’an 271000 Shandong, China; 5 Beijing Botanical Garden, Institute of Botany, Chinese Academy of Sciences, Beijing 100093, China

Correction to: *Horticulture Research*https://doi.org/10.1038/s41438-021-00658-0 published online 01 November 2021

In the originally published version of this manuscript, there was an error in the CACMS Innovation Fund grant number. The grant number should read “CI2021A04117” instead of “C12021A04117”. This error has been corrected.

